# Loss of EIF4G2 mediates aggressiveness in distinct human endometrial cancer subpopulations with poor survival outcome in patients

**DOI:** 10.1038/s41388-024-02981-x

**Published:** 2024-02-22

**Authors:** Sara Meril, Maya Muhlbauer Avni, Chen Lior, Marcela Bahlsen, Tsviya Olender, Alon Savidor, Judit Krausz, Hila Belhanes Peled, Hila Birisi, Nofar David, Shani Bialik, Ruth Scherz-Shouval, Yehuda Ben David, Adi Kimchi

**Affiliations:** 1 Department of Molecular Genetics, Weizmann Institute of Science, Rehovot, 7610001 Israel; 2 Department of Obstetrics and Gynecology, Emek Medical Center, Afula, Israel; 3 The Ruth and Bruce Rappaport Faculty of Medicine, Technion, Haifa, Israel; 4 Department of Biomolecular Sciences, Weizmann Institute of Science, Rehovot, 7610001 Israel; 5 The de Botton Institute for Protein Profiling of the Nancy and Stephen Grand Israel National Center for Personalized Medicine (G-INCPM), Weizmann Institute of Science, Rehovot, 7610001 Israel; 6 Pathology Department, Emek Medical Center, Afula, Israel

**Keywords:** Endometrial cancer, Cancer screening, Cancer therapy

## Abstract

The non-canonical translation initiation factor EIF4G2 plays essential roles in cellular stress responses via translation of selective mRNA cohorts. Currently there is limited and conflicting information regarding its involvement in cancer development and progression. Here we assessed its role in endometrial cancer (EC), in a cohort of 280 EC patients across different types, grades, and stages, and found that low EIF4G2 expression highly correlated with poor overall- and recurrence-free survival in Grade 2 EC patients, monitored over a period of up to 12 years. To establish a causative connection between low EIF4G2 expression and cancer progression, we stably knocked-down EIF4G2 in two human EC cell lines in parallel. EIF4G2 depletion resulted in increased resistance to conventional therapies and increased the prevalence of molecular markers for aggressive cell subsets, altering their transcriptional and proteomic landscapes. Prominent among the proteins with decreased abundance were Kinesin-1 motor proteins, KIF5B and KLC1, 2, 3. Multiplexed imaging of the EC patient tumor cohort showed a correlation between decreased expression of the kinesin proteins, and poor survival in patients with tumors of certain grades and stages. These findings reveal potential novel biomarkers for Grade 2 EC with ramifications for patient stratification and therapeutic interventions.

## Introduction

The non-canonical translation initiation factor EIF4G2 (also known as DAP5/p97/Nat1 [1–4]) plays essential roles in cellular stress responses via translation of selective mRNA cohorts. As a scaffold protein that recruits the 40S ribosome to the 5’ end of the mRNA, EIF4G2 drives translation independently of the mRNA’s 5’cap structure and/or canonical cap-binding proteins through various alternative mechanisms [5–9]. Importantly, EIF4G2 is necessary for embryonic development at early stages [10–12] and differentiation of both human and mouse embryonic stem cells (hESC, mESC) [13, 14]. Among EIF4G2’s mRNA targets are pro- and anti-apoptotic proteins that are specifically translated during apoptosis [15–17] and mitosis [18], an N-terminally truncated isoform of p53 that is translated during cell stress [19], and epigenetic modulators such as HMGN3 and KMTD2 that are essential for hESC differentiation [14, 20]. These indicate an important role for EIF4G2 in the translation of factors critical for cell fate decisions in somatic and embryonic stem cells, and it is not surprising that it has been linked to cancer.

Paradoxically, a limited number of studies have attributed both oncogenic and tumor-suppressive capabilities to EIF4G2. In triple negative metastatic breast cancer, EIF4G2 was shown to mediate non-canonical translation of targets involved in cell migration, the epithelial-mesenchymal transition (EMT) and invasion, and its overexpression correlated with and promoted tumor metastasis [21]. Likewise, a correlative study in gastric cancer showed that increased *EIF4G2* mRNA expression was associated with poor prognosis [22]. On the other hand, *EIF4G2* expression was reduced in bladder cancer, correlating with tumor de-differentiation and invasiveness [23]. Our recent analysis of the TCGA database demonstrated a significant reduction in *EIF4G2* mRNA expression in 7/24 primary tumor types compared to healthy tissue, as opposed to 2 tumor types that showed significant increased mRNA expression [24]. Moreover, we identified deleterious mutations and significantly occurring somatic missense mutations in *EIF4G2* in various cancers, several of which were proven to be loss-of-function. Thus, it appears that EIF4G2’s contribution to cancer varies according to the type and/or stage. Notably, endometrial cancer (EC) was prominent among the cancers showing reduced *EIF4G2* expression and the presence of somatic mutations, representing 14% of all occurrences of the mutations among all examined cancer types [24].

EC is the most common gynecological cancer in developed countries, with increasing incidence in recent years due to the rising prevalence of obesity, a main risk factor for the disease [25]. EC staging according to the International Federation of Gynecology and Obstetrics (FIGO) indicates the degree of invasiveness, with stage 1 limited to the corpus uteri and stages 2–4 progressively involving increased invasiveness and distant metastases. FIGO also grades tumors on a scale of 1–3 according to the relative proportions of the glandular and solid-tumor components. Grade 1 is well differentiated, Grade 2 is considered moderately differentiated, and Grade 3 is poorly differentiated, with a solid-tumor component less than 6%, 6–50%, and more than 50%, respectively. Buckham’s dualistic classification combines several factors, among them histological sub-type, grade, and hormone dependency into Type-1 and Type-2 EC. Type-1 hormone-dependent endometrioid cancer affects approximately 80% of patients, mostly classified as low grade (Grades 1,2 endometrioid adenocarcinoma) with favorable prognosis (5-year overall survival (OS) rate, 85%). Type-2 EC, diagnosed in the remaining 20%, is characterized by hormone-independent, high-grade (Grade 3) endometrioid adenocarcinomas, serous clear cell, carcinosarcomas and undifferentiated tumors, with a higher risk of metastasis and a poorer prognosis (5-year OS, ~55%) [26].

Standard treatment for EC is surgical resection, and depending on tumor stage and grade, is often accompanied by adjuvant therapy such as radiation and chemotherapy [26]. While survival rates for patients with low grade, early detected tumors can be as high as 95%, high grade and recurrent tumors are more refractory to treatment, and overall survival rates drop to 15–17% [27]. Thus, there is still a pressing need for new and advanced therapies and prognostic markers for the successful treatment of EC. One promising approach involves specifically targeting aggressive subpopulations with stem-like characteristics that can be identified by expression of certain markers such as CD133, CD44 and ALDH1A1 [27]. These cancer sub-populations, which have been identified in reproductive cancers such as breast, EC and various other cancers types, have been shown to acquire increased metastatic capacity and invasiveness, increased resistance to chemotherapies and radiotherapies, and increased tumorgenicity. As their presence is presumed to confer tumor aggressiveness, strategies to reduce their growth or induce their differentiation have been recently promoted [28].

Here we assessed the contribution of EIF4G2 to EC and determined that low protein expression in a cohort of 280 EC patients was associated with decreased overall survival of patients with Grade 2 EC. The functional significance of low EIF4G2 expression was assessed in HEC-1A and RL95-2 EC knock-down (KD) cell lines, which showed increased resistance to Taxol and radiation treatment, and enrichment for cells with high expression of CD133 and CD44 and altered transcriptomic and proteomic signatures. Among the proteins with decreased abundance were direct translation targets of EIF4G2, such as the kinesin-1 motor protein, which likewise showed a correlation between decreased protein expression and decreased overall survival rates in patients with more advanced EC. Our results show the prognostic potential of EIF4G2 and its potential protein targets.

## Results

### Low protein expression of EIF4G2 correlates with poor prognosis in endometrial cancer patients

**Fig. 1:**
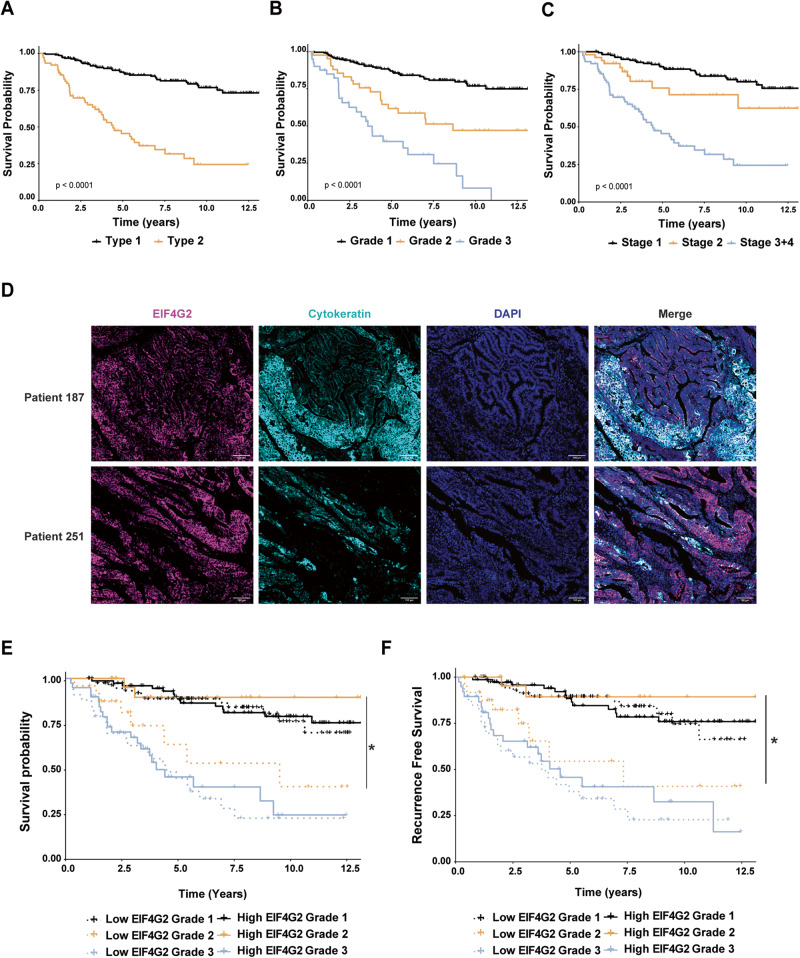
Low EIF4G2 expression correlates with poor prognosis in Grade 2 EC patients. Overall survival of 280 endometrial patients according to tumor (**A**) Type, (**B**) grade and (**C**) stage. Kaplan–Meier *P*-val was determined using log rank P test. **D** formalin fixed paraffin embedded (FFPE) tumor microarray (TMA) sections from the patient cohort were co-immunostained for EIF4G2, CK and DAPI. Representative images of two patients are shown. Scale bar 100 µM. **E**, **F** EIF4G2 staining within CK positive cells was stratified according to high (above median staining intensity) and low (below median staining intensity) levels and overall survival (**E**) and recurrence free survival (**F**) were assessed by Kaplan–Meier analysis. Log rank *P*-val was calculated from paired comparisons with FDR correction. *p < 0.05.

The clinical significance of changes in expression of EIF4G2 in EC was assessed in a cohort of 280 EC patients collected and followed for up to 12 years post-surgery. Details on patient data are provided in Supplementary Table 1. All underwent surgical resection of the uterus, with or without adjuvant treatment. Kaplan–Meier analysis of OS of the samples recapitulated the expected patient outcomes based on type, grade, and stage of EC, with Type-2 and more advanced grades and stages showing lower survival rates (Fig. 1A–C).

Tumor microarrays (TMA) were generated with cores derived from the 280 re-sectioned formalin-fixed paraffin embedded (FFPE) tumors. Each TMA was immunostained by multiplex immunofluorescence imaging for EIF4G2 and cytokeratin (CK), an epithelial marker used to identify the epithelial tumor cells, and staining intensity was only quantified in CK positive cells (Fig. 1D). Low and high EIF4G2 expression levels were defined as values below and above the median expression level, respectively (Supplementary Table S1). Expression levels were then correlated with patient survival by Kaplan–Meier survival analysis. Overall, there was no statistically significant difference in OS between patients exhibiting lower or higher intensity of EIF4G2 protein staining, although there was a slight trend towards lower survival in the low expressers (Supplementary Fig. S1A). Similarly, OS and recurrence-free survival (RFS) did not differ for patients with high or low EIF4G2 expression when analyzed by tumor type (Supplementary Fig. S1B, C) or tumor stage (Supplementary Fig. S1D, E). However, differences in EIF4G2 expression became statistically meaningful when patients were stratified by tumor grade; there was significantly lower OS and RFS in patients with Grade 2 tumors that expressed low levels of EIF4G2 (Fig. 1E, F). Also, low levels of EIF4G2 expression were associated with increased recurrence rates (Table 1). These differences could not be attributed to different treatments received following diagnosis, and the majority of both high and low expressing tumors were classified as low stage (Table 1), meaning the extent of migration and invasion was limited in both groups. Collectively, these data reveal that lower EIF4G2 expression is associated with poorer outcome in EC based on grade, with an especially strong association between low EIF4G2 expression and poor OS and RFS in Grade 2 EC patients.

**Table 1:** Grade 2 patient parameters stratified by low and high EIF4G2 protein Expression.

		EIF4G2 low (Intensity < 3.7)	EIF4G2 high (Intensity > 3.7)
Patient data	Number of patients	25	26
	Age	65.4	62.5
	BMI	29.0	42.6
Stage	1	19 (76%)	19 (73%)
	2	3 (12%)	5 (19.2%)
	3	3 (12%)	2 (7.6%)
	4	0	0
Therapies	Received therapy overall	17 (68%)	19 (73%)
	Chemotherapy	4/17 (23.5%)	1/19 (5.2%)
	Brachytherapy	15/17 (88.2%)	18/19 (94.7%)
	External radiation	7/17 (41.1%)	5/19 (26.3%)
Survival statistics	Recurrence	6 (24%)	4 (15.3%)
	Overall survival (months)	48.6	67.1
Number of patients from date of surgery	1 year	3	0
	2–3 years	12	8
	4–5 years	1	5
	6–7 years	1	2
	8–12 years	8	11

### EIF4G2KD results in increased resistance to anti-cancer agents in HEC-1A and RL95-2 EC cell lines

**Fig. 2:**
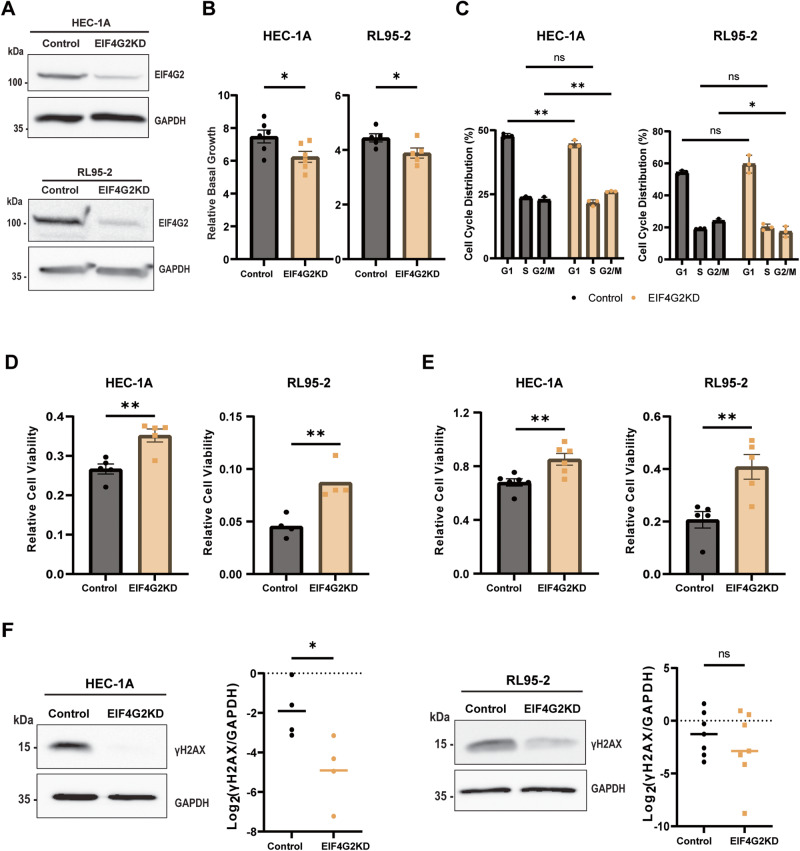
Depletion of EIF4G2 in HEC-1A and RL95-2 cells results in increased therapy resistance. **A** Total cell lysate form HEC-1A (upper panel) and RL95-2 (lower panel) control and EIF4G2KD cells were subjected to western blot analysis for EIF4G2 and GAPDH as loading control. Representative blots are shown. **B** Relative basal growth of control and EIF4G2KD cells was measured by CellTiter-Glo assay in HEC-1A (left) and RL95-2 (right). Relative growth was calculated as luminescence of cells at day 4 (T4) relative to corresponding cells at day 0 (T0). Shown are the mean relative luminescence values ± SEM of n > 5 independent experiments. Significance was determined by two tailed t-test. *p < 0.05. **C** Cell cycle distribution of HEC-1A (left) and RL95-2 (right) control and EIF4G2KD cells after 24 h in culture. Shown are the % total population in each stage, expressed as mean ± SEM of 3 independent experiments. Statistical significance was determined by one way ANOVA. *p < 0.05; **p < 0.01; ns: not significant. **D** Cell viability was measured by CellTiter-Glo assay in HEC-1A (left) and RL95-2 (right) control and EIF4G2KD cells incubated with DMSO or 2.5 nM Taxol for 4 d. Luminescence values of Taxol treated samples were normalized to the values of corresponding DMSO treated samples, representing relative viability. Shown are the mean relative luminescence values ± SEM of *n* > 4 independent experiments. Significance was determined by two tailed t-test. **p < 0.01. **E** HEC-1A (left) and RL95-2 (right) control and EIF4G2KD cells were exposed to either 16 Gy or 8 Gy X-ray irradiation, respectively. Cell viability was assayed 4d later as in **D** relative to mock irradiated cells. Shown are the mean relative luminescence values ± SEM of *n* > 5 independent experiments. Significance was determined by two tailed t-test. **p < 0.01. **F** Total cell lysates form HEC-1A (left) and RL95-2 (right) (**E**) control and EIF4G2KD cells were subjected to western blot analysis for γH2AX and GAPDH, as loading control, 4d following irradiation. γH2AX signal was normalized to GAPDH and quantification results are represented as individual data points and also as mean values of 4 (HEC-1A) or 7 (RL95-2) independent experiments.

To establish a causative link between EIF4G2 and tumor aggression, EIF4G2 was depleted in the HEC-1A and RL95-2 EC cell lines by viral infection with vectors expressing shEIF4G2 or shGFP as control (Fig. 2A). HEC-1A cells were derived from a Stage 1A moderately well differentiated adenocarcinoma [29], and have been classified histologically as Grade 2 [30]. RL95-2 cells were derived from Grade 2 moderately differentiated adenosquamous carcinoma [31]. Depletion of EIF4G2 reduced the basal growth rates compared to the control cells in both HEC-1A and RL95-2 cells (Fig. 2B). Only minimal differences of uncertain biological significance were observed in cell cycle distribution (Fig. 2C, Supplementary Fig. S2A–D). We then tested the effect of EIF4G2 depletion on the responses to Taxol (Paclitaxel), the common chemotherapy agent used against aggressive EC tumors, and X-ray irradiation, a front-line therapy given either internally (brachytherapy) or externally [32]. While RL95-2 cells were more responsive to the treatments than HEC-1A cells, in both cell types EIF4G2KD cells exhibited increased viability following treatment with Taxol or irradiation compared to control cells (Fig. 2D, E). Western blotting for γH2AX, a histone variant that is phosphorylated in response to double stranded DNA breaks and serves as a marker for DNA damage and resolution, showed that in both cell lines, EIF4G2KD cells led to reduced levels of γH2AX compared to the control cells (Fig. 2F, Supplementary Fig. S2E). This suggests that the irradiated EIF4G2KD cells either underwent less DNA damage or managed to resolve the damage faster and/or more efficiently compared to control irradiated cells. Overall, EC cells that are depleted of EIF4G2 displayed reduced sensitivity to standard therapies used clinically to treat EC.

**Fig. 3:**
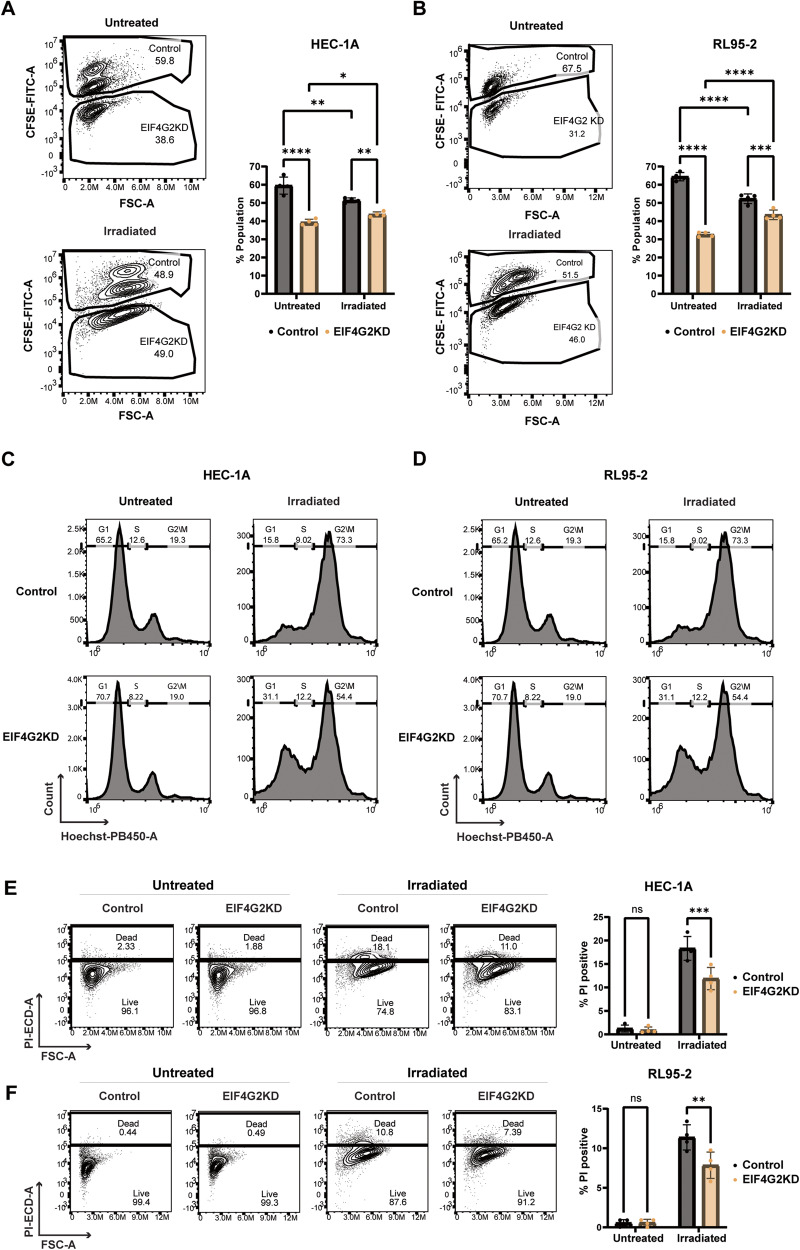
Depletion of EIF4G2 in HEC-1A and RL95-2 cells attenuates cell cycle arrest and cell death following irradiation, leading to enrichment upon co-culture with control cells. Control HEC-1A or RL95-2 cells were stained with CFSE and co-cultured with equal quantities of EIF4G2KD cells. 24 h later, they were irradiated with 16 Gy (HEC-1A) or 8 Gy (RL95-2) X-rays, or left untreated, and analyzed 72 h later by flow cytometry. HEC-1A (**A**) or RL95-2 (**B**) co-cultures were gated based on CFSE staining to identify control and EI4FG2 KD cells. Shown are representative density plots; the percentage of each cell type within the total live cell population is shown in the graphs, as mean ± SEM of 4 independent experiments. Statistical significance was determined by two-way ANOVA. (*p < 0.05, **p < 0.01, ***p < 0.001, ****p < 0.0001). **C**, **D** Cell cycle distribution was determined after staining with Hoechst for DNA content for CFSE+ (control) and CFSE− (EIF4G2KD) populations in HEC-1A (**C**) or RL95-2 (**D**) cells. Shown are representative histograms from one of 3 independent experiments. HEC-1A (**E**) and RL95-2 (**F**) cells were stained with PI as a measure of cell death and the percentage of PI positive (dead) cells within CFSE+ and CFSE− populations was determined. Shown are representative density plots of the gated populations, and graphs showing the mean ± SEM of 4 independent experiments. Statistical significance was determined by two-way ANOVA (**p < 0.01, ***p < 0.001, ns not significant).

The increased resistance to therapies observed suggests that reduced expression of EIF4G2 should confer a survival advantage, resulting ultimately in their persistence and enrichment within a mixed population of cells. To test this, control HEC-1A cells were stained with CFSE, and mixed together in equal quantities with unstained EIF4G2KD cells in co-cultures. 24 h later, the cells were irradiated or left untreated as above, and analyzed by flow cytometry for cell viability (Propidium Iodide (PI) uptake) and cell cycle distribution after an additional 72 h (Supplementary Fig. S3, gating strategy). In untreated cells, 4d total culture resulted in a population shift favoring the control CFSE+ cells in HEC-1A cells, and even more dramatically in RL95-2 cells (Fig. 3A, B), likely due to the higher growth rates observed above (Fig. 2B). In contrast, 72 h following irradiation, the proportion of EIF4G2KD cells within the population increased, approaching the original equal levels (Fig. 3A, B). Irradiation resulted in pronounced G2/M arrest and cell death in control cells in both cell lines, with the death response more pronounced in HEC-1A cells (Fig. 3C–F). Significantly, the proportion of cells arrested in G2/M was lower in EIF4G2KD cells, suggesting that depletion of EIF4G2 resulted in escape from cell cycle arrest. In addition, EIF4G2 depletion resulted in a 1.5-fold lower cell death rate compared to control HEC-1A cells, with a smaller effect on cell death in the RL95-2 cells (Fig. 3E, F). Thus, the relative enrichment in EIF4G2KD cells within the co-culture following irradiation most likely resulted from the combination of decreased cell death and cell cycle arrest in EIF4G2KD cells.

### EIF4G2 depletion increases the proportion and treatment resistance of cells expressing markers of aggression CD133 and CD44

**Fig. 4:**
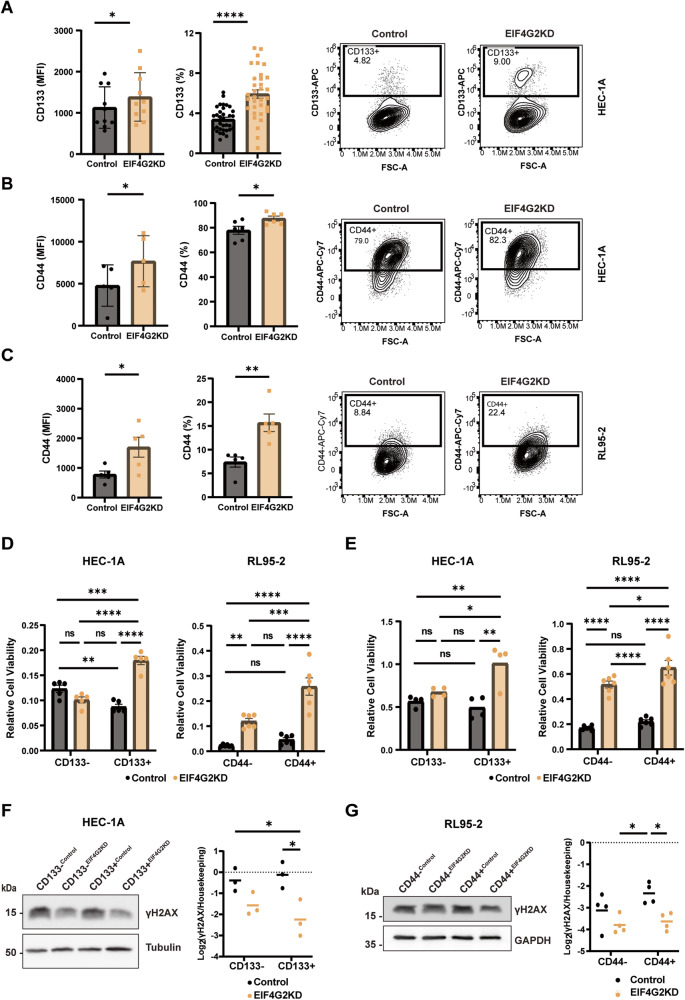
EIF4G2KD increases CD133 and CD144 expression and viability of CD133+ and CD44+ populations following therapy in HEC-1A and RL95-2 cells. Flow cytometry analysis of cell-surface expression of CD133 (**A**) and CD44 (**B**, **C**) in control and EIF4G2KD HEC-1A cells (**A**, **B**) and control and EIF4G2KD RL95-2 cells (**C**). Quantification of results is presented as mean ± SEM, *n* > 5 for both overall mean florescent intensity (MFI) and as percent of total population. *P*-vals were calculated using two-tailed t-test (*p < 0.05, **p < 0.01, ****p < 0.0001). A representative flow cytometry plot is shown for each. **D** Control and EIF4G2KD HEC-1A and RL95-2 cells were separated for CD133 and CD44 expression, respectively. Separated populations were treated with 2.5 nM Taxol or DMSO as control for 4 d and cell viability assayed by CellTiter-Glo. Luminescence was normalized to DMSO treated cells. Left graph, HEC-1A cells, right graph RL95-2 cells. **E** Cell viability of HEC-1A (left) and RL95-2 (right) CD133+ and CD133− or CD44+ and CD44- control and EIF4G2KD separated populations 4 d after exposure to 16 Gy (HEC-1A) or 8 Gy (RL95-2) X-ray irradiation. For **D**, **E**, results are presented as mean values ± SEM of *n* > 4 independent experiments. Significance was determined by two-way ANOVA. (*p < 0.05, **p < 0.01, ***p < 0.001, ****p < 0.0001). Total cell lysates form HEC-1A (**F**) and RL95-2 (**G**) CD133+ and CD133- or CD44+ and CD44- control and EIF4G2KD separated populations were subjected to western blot analysis for γH2AX and GAPDH, as loading control 4 d following irradiation. γH2AX signal was normalizedC to GAPDH and quantification results are presented as individual data points and also as mean values of 4 independent experiments. Statistical significance was determined for log_2_ transformed mean values by two-way ANOVA (*p < 0.05).

In order to further characterize the effect of EIF4G2 protein depletion on cancer outcome, and to understand why EIF4G2KD cells are more resistant to therapies, we examined the prevalence of aggressive sub-populations within the control and EIF4G2KD cells by probing for CD133 (PROM1) and CD44. FACS analysis for cell surface expression of these markers (see Supplementary Fig. S4A, B for gating strategy) indicated that EIF4G2KD increased both markers’ expression intensity and the population distribution of positive cells (Fig. 4A, B). Similar, yet more modest, results were also observed upon transient siRNA transfection (Supplementary Fig. S4C, D). RL95-2 cells did not express CD133, even upon EIF4G2KD, yet EIF4G2 depletion did increase CD44 surface expression intensity by two-fold (Fig. 4C), similar to the HEC-1A cells.

The potentially more aggressive sub-populations were separated from the KD cells using anti-CD133 and anti-CD44 conjugated magnetic beads for HEC-1A and RL95-2 cells, respectively. The percentage of the CD133^+^ and CD133^−^ populations remained stable over time in the separated HEC-1A populations (Supplementary Fig. S4E), and there was no significant difference in their proliferation rates (Supplementary Fig. S4F). Cell viability of the separated control and EIF4G2KD cells was then assessed following treatments with Taxol and X-ray irradiation. CD133+^EIF4G2KD^ HEC-1A cells and CD44+^EIF4G2KD^ RL95-2 cells displayed significant increases in viability compared to all other separated populations in response to Taxol treatment (Fig. 4D). Similarly, the relative number of viable cells in the CD133+^EIF4G2KD^ HEC-1A cells was almost double that of the other cell populations, and the viable CD44+^EIF4G2KD^ RL95-2 cells were almost three times more than the control populations (Fig. 4E). As expected, western blotting indicated that γH2AX was activated, yet remained significantly lower in EIF4G2KD CD133+ HEC-1A and CD44+ RL95-2 cells (Fig. 4F, G). This implies that DNA damage was reduced and/or more quickly resolved in the EIF4G2KD cells, and thus these more aggressive sub-populations were more likely to survive irradiation.

### Downregulation of EIF4G2 expression alters the transcriptomic and proteomic signature of CD133+ cells and selectively impairs translation of kinesin 1 proteins

**Fig. 5:**
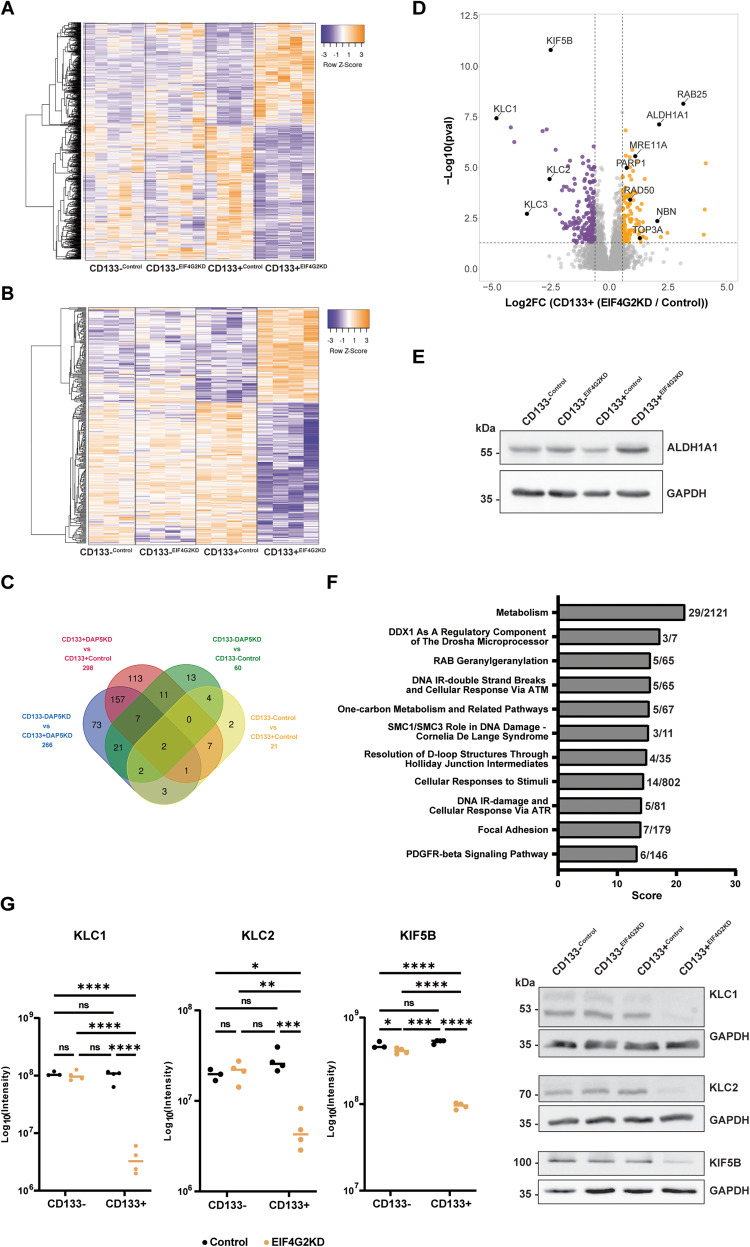
EIF4G2KD alters the transcriptomic and proteomic signatures of CD133+ population in HEC-1A cells. Control and EIF4G2KD HEC-1A cells were FACS sorted by CD133 marker expression and subjected to RNA-seq and MS analysis. **A** Heat map showing hierarchical clustering of gene expression levels of all DEGs identified following RNA-seq analysis of CD133- and CD133+ sorted control and EIF4G2KD populations. **B** Heat map showing hierarchical clustering of all proteins with significantly changed abundance in MS analysis of CD133− and CD133+ sorted control and EIF4G2KD HEC-1A cells. **C** Venn diagram showing overlap of all differentially abundant proteins among the four comparisons. Numbers at the edges of the diagram represent the overall number of proteins with differential abundance in each comparison. **D** Volcano plot of the Log_2_ (Fold Ratio) of the abundance of the detected proteins in CD133+^EIF4G2KD^/CD133+^Control^ comparison, vs. their significance expressed as log_10_ *p*-value. Proteins with significant increased abundance are indicated in orange, and decreased abundance in purple. **E** Total cell lysates from separated control and EIF4G2KD HEC-1A populations were subjected to western blot analysis for ALDH1A1 and GAPDH as loading control. Shown is a representative blot of 3 independent experiments. **F** High scoring significant pathways identified by GeneAnalytics pathway analysis of the set of proteins with increased abundance in the CD133+^EIF4G2KD^/CD133+^Control^ comparison. Score numbers on X-axis indicate significance, numbers at right represent the number of proteins identified in the dataset out of the total number of proteins within the given pathway. **G** Protein expression of KLC1, KLC2 and KIF5 based on the abundance detected by the MS analysis. Data presented as mean of Log_10_ (Intensity), *n* = 4. Statistical significance was determined by two-way ANOVA (*p < 0.05, ***p < 0.001, ****p < 0.0001). Representative western blots of HEC-1A separated CD133− and CD133+ control and EIF4G2KD cells validating the MS results for KLC1, KIF5B, and KLC2. GAPDH was used as a loading control. Shown is one representative blot of *n* = 3.

In order to better understand the phenotypic differences in response to therapies, control and KD HEC-1A populations were FACS sorted and then subjected to RNA-seq analysis and mass spectrometry (MS) analysis. The CD133− and CD133+ control populations showed relatively minor changes in their transcriptomic profiles with only 77 differentially expressed genes (DEGs) (Supplementary Table S2). Annotation by GeneAnalytics [33] of this set indicated enrichment in pathways associated with “cytoskeletal signaling” (7 genes) and “embryonic and induced pluripotent stem cell and lineage specific markers” (5 genes: SOX17, TNNI3, PAX2, PROM1 (CD133), FGF18) (Supplementary Fig. S5A), consistent with a more stem-like phenotype in the CD133+ cells. There was also a high degree of similarity between the transcriptomic profiles of the CD133− populations regardless of EIF4G2 status (137 DEGs, with no high scoring pathways by GeneAnalytics), indicating little effect of EIF4G2 depletion on the general HEC-1A cells. In contrast, the RNA-seq analysis highlighted a strong transcriptomic difference between the CD133+^EIF4G2KD^ cells and all other populations (Fig. 5A). In other words, EIF4G2 depletion had the largest effects on gene expression in the context of the CD133+ cells, with 939 DEGs between the EIF4G2KD and control CD133+ populations. Moreover, its depletion amplified the relatively small transcriptomic differences between the CD133− and CD133+ cells by more than 10-fold (77 DEGs vs 894 DEGs in the EIF4G2KD comparison) (Supplementary Table S2). Gene annotation of both sets of DEGs for significant pathways with high score is shown in Supplementary Fig. S5B, C. Notably, upon EIF4G2KD, ALDH1A1, a prominent marker whose up-regulation is associated with enhanced tumor aggressiveness and therapy resistance in various cancers, including EC [34–38], was increased in expression in the CD133+ cells. Thus decreased EIF4G2 expression not only enhanced the prevalence of the aggressive populations (in terms of CD133 and CD44 expression), but also changed their transcriptomic profile compared to CD133+ cells.

The phenotypic and transcriptomic effects of EIF4G2KD on CD133+ cells are likely ascribed to its role as a translation factor, as has been shown in ESCs [14]. Polysome profiling analysis of EIF4G2 and control KD HEC-1A cells showed no changes in global translation (Supplementary Fig. S6A), consistent with EIF4G2’s involvement in selective translation of specific mRNA targets [8, 13, 14, 20]. Furthermore, investigation of the proteome of EIF4G2KD CD133− and + cells by MS confirmed that the majority of proteins were not changed in abundance. An overall comparison of the proteomic signatures of the CD133− and + cells with or without EIF4G2 KD resembled the transcriptomic pattern, in that the CD133+^EIF4G2KD^ proteome was altered compared to the other 3 sorted populations, which all shared a similar profile (Fig. 5B). Enriching for CD133 did not in itself result in major differences in the proteome; only 21 proteins changed in abundance in the control CD133+ vs CD133− populations (Fig. 5C). The difference between the populations was enhanced by EIF4G2 KD, with 266 proteins exhibiting changed abundance in the CD133+^EIF4G2KD^ vs CD133−^EIF4G2KD^ cells. EIF4G2 KD had a moderate effect on the proteome of CD133− cells, with 60 differentially abundant proteins in the KD vs control cells, and a stronger effect on the CD133+ cells, with 298 proteins with differential abundance identified when comparing CD133+^EIF4G2KD^ and CD133+^Control^ populations (Fig. 5C). Thus, EIF4G2KD affected both the transcriptomic and proteomic profiles of the CD133+ cells to the greatest extent.

Of the 298 proteins with differential abundance in the CD133+^EIF4G2KD^ and CD133+^Control^ comparison, 20 were also observed to change in the CD133−^EIF4G2KD^ and CD133−^Control^ comparison, indicating a dependence on EIF4G2 expression unrelated to the cell population. The majority of changes, however, occurred due to both the CD133 and the EIF4G2KD status. Of the 298 differentially abundant proteins in the CD133+ control and KD cells, 177 showed decreased abundance and 121 were increased. Pathway analysis of the decreased set of proteins by GeneAnalytics showed enrichment in pathways associated with the mitochondrial electron transport chain, metabolism, cell adhesion, actin nucleation and G-protein/Rho GTPase signaling pathways (Supplementary Fig. S6B).

The MS analysis showed a set of 121 proteins with increased abundance specifically in the EIF4G2KD CD133+ cells compared to the control CD133+ cells (Fig. 5D). While these do not represent potential EIF4G2 mRNA translation targets, they do shed light on the indirect contribution of EIF4G2 to EC aggressiveness, particularly within CD133+ cells. As observed for its mRNA, the tumor aggressiveness marker ALDH1A1 increased more than 4-fold in the MS analysis (Fig. 5D). This increase was validated by western blotting (Fig. 5E). No difference was observed between control CD133− and + cells in either the MS dataset or the western blot. GeneAnalytics pathway analysis of the 121 proteins with increased abundance showed they were enriched in the general pathway “metabolism” and several related pathways with the same 3–5 proteins involved in DNA repair of double strand breaks and response via ATM (Fig. 5F). These included PARP1, RAD50, MRE11A, TOP3A and NBN (also known as NBS1) (Fig. 5D).

Of the 177 proteins with decreased abundance, 126 did not show comparable decreases in mRNA expression (Supplementary Fig. S6C), indicating that the decreased protein levels observed were not due to changes in gene transcription or mRNA stability. While not excluding other factors that can influence protein steady state levels, this set of proteins may represent potential translation targets of EIF4G2. In fact, several of these proteins have been previously identified as EIF4G2 translation targets by unbiased screens using either ribosome footprinting [8, 13, 20] or polysome profiling [14] in additional cell types. These included KIF5B [8], which was among the highest scoring proteins with changed abundance in the EIF4G2KD CD133+ cells. KIF5B is a kinesin heavy chain of the kinesin-1 microtubule-based motor protein. Notably, 2 of the light chains often associated with kinesin-1, KLC1 and KLC2, also decreased in abundance upon EIF4G2KD in the CD133+ cells (Fig. 5D), and a third light chain, KLC3, showed reduced abundance upon EIF4G2KD in both CD133− and CD133+ cells. The MS results were confirmed by western blot analysis of these proteins; KLC1, KLC2 and KIF5B steady state levels strongly decreased in the CD133+^EIF4G2KD^ population compared to all others (Fig. 5G). Thus, EIF4G2 is necessary to maintain the steady state levels of kinesin-1 in HEC-1A CD133+ cells.

### Low protein staining intensity of EIF4G2 dependent targets KLC1 and KIF5B correlates with poor patient survival

**Fig. 6:**
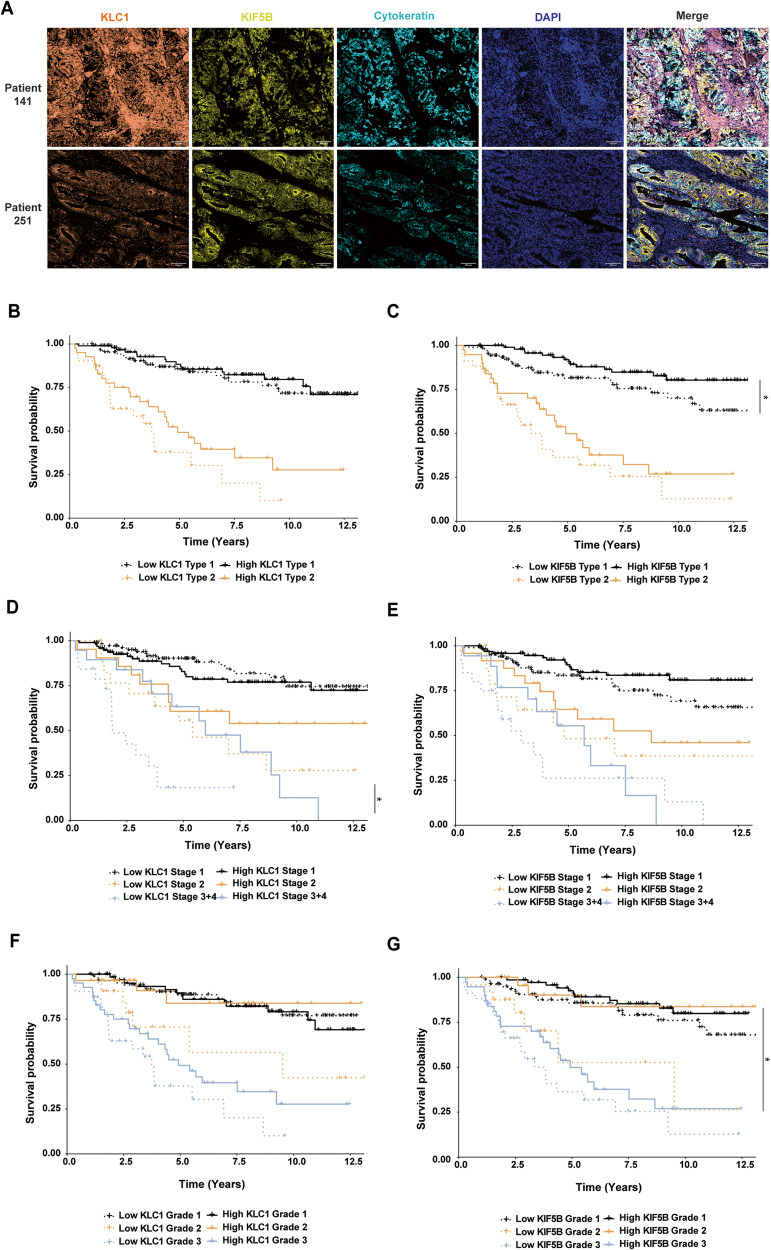
Low protein expression of EIF4G2 targets KLC1 and KIF5B correlates with worse overall survival in EC patients. **A** FFPE tumor TMA sections from the 280 patients were immunostained for KLC1, KIF5B, CK and DAPI. Representative images of two patients are shown. Scale bar 100 µM. Overall survival of the 280 endometrial patients according to tumor (**B**, **C**) type, (**D**, **E**) stage, and (**F**, **G**) grade. KLC1 (**B**, **D**, **F**) and KIF5B (**C**, **E**, **G**) staining in CK positive cells was stratified according to high and low intensity levels compared to the calculated median, and overall survival was assessed by Kaplan–Meier statistics. Log rank *P*-val was determined for protein expression of each of these signatures. Paired comparison was calculated with FDR correction. *p < 0.05, ***p < 0.001.

The status of the kinesin-1 motor proteins was assessed in EC patients. Sequential sections from the TMAs of the patient cohort used in Fig. 1 were stained for KLC1 and KIF5B together with CK by multiplex immunofluorescence (Fig. 6A). Expression levels based on quantification of staining intensity were compared to EIF4G2 staining of the next section from the same TMA cores. Notably, there was a small but significantly positive correlation between expression of EIF4G2 and KLC1, and a moderately larger positive correlation with KIF5B expression (Supplementary Fig. S7A, B). OS was then correlated with expression of KLC1 and KIF5B, with low and high expression defined as values that were less than or greater than the median staining intensity for each protein, respectively. In general, OS curves trended lower in patients with low KLC1 or low KIF5B expressing tumors upon stratification of tumors by type, grade and stage, although this trend was not always statistically significant (Fig. 6B–G). Notably, OS was significantly reduced in Type 1 and Grade 2 tumors with low KIF5B staining intensity (Fig. 6C, G), and in low KLC1 expressing tumors of advanced Stages 3/4 (Fig. 6D). The shift in patient survival probability in Grade 2 tumors expressing low KIF5B (and also KLC1, although this was not statistically significant) towards the Grade 3 curve was reminiscent of the OS curve of Grade 2 low expressing EIF4G2 patients (Figs. 1E and 6F,G). RFS analysis (Supplementary Fig. S7C–H) revealed a significant decrease in Grade 2 patients with low KLC1 expression, and in Type 1 and Grade 2 patients with low KIF5B staining intensity. Overall, these results show a strong correlation between low protein expression of either KLC1 or KIF5B and poorer survival outcomes in specific EC tumor classes.

## Discussion

Here we examined the contribution of the non-canonical translation factor EIF4G2 to endometrial cancer in human patients and cell lines. EC cells depleted of EIF4G2 were less sensitive to Taxol and radiation, common therapeutic agents used clinically to treat EC. Furthermore, EIF4G2 KD resulted in enrichment in cells expressing higher levels of markers such as CD133, CD44 and ALDH1A1 associated with aggressiveness, often referred to as cancer stem cells (CSC). This sorted cell population had a vastly changed transcriptome and proteome upon EIF4G2 depletion, indicating an inherently different cell phenotype, and was particularly resistant to both chemotherapy and radiotherapy. The resistance to therapy is consistent with the increased ALDH1A1 expression at both the mRNA and protein levels. Specifically, ALDH1A1 overexpression was shown to be necessary for Paclitaxel resistance in EC cells grown as spheroids in vitro [35]. The resistance to radiation may be connected to increased expression of several DNA damage repair proteins, including PARP1, and components of the MRN (MRE11, RAD50, and NBN) complex that senses and repairs double stranded DNA breaks. In fact, lower levels of γH2AX in EIF4G2KD cells are consistent with an improved ability to resolve DNA damage. Thus, KD cells are better equipped to deal with DNA damage and are more likely to survive DNA damaging treatments. This infers a survival advantage to tumor cells that display lower expression levels of EIF4G2 expression, with implications for enhanced drug resistance and/or recurrence.

Overall, the data predict that loss of or decreased EIF4G2 expression, which has been previously documented in EC [24], would significantly lead to poorer clinical outcome. Notably, our analysis of EIF4G2 protein levels in the EC tumors indicated that low expression correlated with poorer outcome and increased recurrence rates specifically in patients with Grade 2 tumors. This is consistent with EIF4G2’s previously reported divergent association with different types and stages of cancers [21–24]. Importantly, Grade 2 EC tumors are usually considered low risk and are not treated aggressively, yet some manifest clinical indications more reminiscent of higher Grade 3 tumors and should be treated as such. EIF4G2 appears to be a promising prognostic marker to differentiate between non-aggressive and more aggressive Grade 2 tumors. Based on our data, Grade 2 tumors with low EIF4G2 levels would benefit from more aggressive adjuvant treatment. In fact, while most of the Grade 2 patients in our cohort were treated with radiation therapy, only a small number received chemotherapy. Considering the relative resistance of EIF4G2 depleted EC cells to Taxol and radiation in vitro, even when these treatments were applied, they may have been limited in their efficacy in tumors with low EIF4G2 expression. Further assessment of the drug response to these different treatments in tumors with varying levels of EIF4G2 is mandated to clarify the clinical implications of these results.

Our findings on the tumor suppressive nature of EIF4G2 in EC contradict previous in-depth analysis of EIF4G2 function in metastatic triple negative breast cancer, in which EIF4G2 was shown to promote metastasis through translation of factors associated with EMT, motility, angiogenesis and cell survival [21]. Moreover, EIF4G2 protein levels were elevated in metastatic tumors compared to non-metastatic tumors from a very small cohort of triple negative breast cancer patients, and high mRNA levels correlated with poor metastasis-free survival rates [21]. Our much wider protein analysis of a larger group of primary tumors surgically extracted from EC patients did not reveal adverse survival probabilities associated with increased EIF4G2 protein expression, but rather the opposite. In addition to the fact that immunostaining for EIF4G2 protein in tissue is a more accurate description of the relative levels of EIF4G2 specifically within the tumor tissue compared to measurement of total mRNA levels, the discrepancy in the data is consistent with EIF4G2 having different functions in different cancer subtypes. In breast cancer too, EIF4G2 depletion affected metastasis but not growth of primary tumors upon injection of EIF4G2KD cells into mice [21]. Thus EIF4G2’s role, and by extension its direct translation targets, appears to be context dependent, with divergent roles in metastatic and primary tumors.

While our study did not directly determine mRNA translation targets of EIF4G2, our combined transcriptomic and proteomic analysis of EIF4G2KD CD133 + EC cells does provide clues to potential targets among proteins with decreased abundance but unchanged mRNA levels. Downregulated pathways in the MS analysis included pathways related to adhesion, cytoskeleton and integrins, in line with some of the translation targets identified in breast cancer. Other potentially relevant pathways with changes were related to the electron transport chain of oxidative respiration and energy metabolism. Eight proteins associated with complexes I, II, III and IV of the electron transport chain as well as additional proteins involved in mitochondrial protein import or morphology, were all reduced in abundance in EIF4G2KD CD133+ cells (see Supplementary Table S3). These results were reminiscent of hESCs, in which mitochondrial oxidative phosphorylation proteins exhibited reduced translation upon EIF4G2KD, accompanied by a defective oxidative respiratory pathway [14]. Pluripotent stem cells are usually more glycolytic, and differentiation is accompanied by increased reliance on mitochondrial oxidative respiration. Thus, impaired oxidative respiration can limit the transition from pluripotency to more differentiated states [39]. In general, cancer cells are known to rely heavily on glycolysis for energy production (referred to as the Warburg effect), and in some cancers, such as lung, colon, breast and ovarian cancers, osteosarcoma and glioblastoma, CSCs are even more glycolytic compared to their more differentiated counterparts [40]. Endometrial cancer spheroid cells with CSC properties also showed enhanced glycolysis that was dependent on high ALDH activity and ALDH1A1 expression [35]. It therefore follows that the predicted downregulation of the oxidative phosphorylation pathway in EIF4G2KD EC cells likely confers a more stem-like phenotype to these cells. This, combined with increased expression of markers CD133, CD44 and ALDH1A, and the enhanced therapy resistance of these cells, suggests that similar to its role in ESCs, EIF4G2 is necessary for the dynamic transition between more aggressive stem-like and more differentiated EC cells.

As a direct consequence of EIF4G2 depletion in the CD133 + EC cells, decreased protein abundance was observed for components of the Kinesin-1 microtubule motor protein, including heavy chain KIF5B and light chains KLC1, 2, 3. One of these, KIF5B, has been shown to be a direct translation target of EIF4G2 [8, 21]. Interestingly, various mitotic kinesin motor proteins are up-regulated in tumors, and as they play an important role in microtubule dynamics during mitosis and cytokinesis, are targets of anti-cancer drug therapy [41]. KIF5B, however, is a non-mitotic member of the kinesin protein superfamily, and either alone or together with its associated light chains, transports organelles such as mitochondria and endosomes towards the plus end of the microtubules. It has also been shown to be involved in microtubule-dependent mobility of DNA with double strand breaks and positioning of the nuclear envelope to facilitate DNA repair [42, 43]. Interestingly, similar to EIF4G2, KIF5B seems to have divergent roles depending on the tumor context; in triple negative metastatic breast cancer cells, KIF5B levels were increased, leading to acquisition of EMT, invasiveness and stem-like phenotype [44, 45], but silencing of KIF5B in epithelial MDCK cells induced EMT, and enhanced invasive and tumorigenic properties [46]. In the former system, KLC1 levels inversely correlated with KIF5B, and was localized to the cytoplasm while KIF5B localized to the nucleus [44], implying that KIF5B partners with other KLCs or functions independently of KLCs in this system within the nucleus to mediate its pro-tumorigenic effects. In our EC TMA samples, in contrast, both KIF5B and KLC1 localized to the cytosol, and their expression positively correlated. Moreover, low expression levels of both proteins were associated with poor OS and RFS outcomes in the EC patients of specific type, grade and stage. Thus, the kinesin-1 motor protein is likely to play a different role in EC than it plays in metastatic breast cancer, and its expression may serve as a novel prognostic marker in EC, similar to EIF4G2 itself. Our functional analysis of EC cells in vitro coupled with expression analysis of a large cohort of EC patients underscores the potential significance of the EIF4G2-kinesin-1 axis for the development of EC.

In conclusion, our findings strongly support the integration of EIF4G2, KIF5B and KLC1 markers into clinical practice for the management of endometrial carcinoma. This strategic inclusion may empower healthcare professionals to tailor prognosis and treatment plans to each patient’s unique profile, ultimately enhancing patient outcomes and the overall quality of care.

## Materials and methods

### Ethics statement

This study was performed in line with the principles of the Declaration of Helsinki. Patients’ samples and data were collected following approval by the Emek Medical Center Institutional Review Board (IRB, protocol no 0043-22-EMC).

### Human patient samples and tissue microarray construction

TMA-containing cores from 280 female endometrial patients, representing all patient specimens collected over a 12-year period, were retrieved from the archives of HaEmek Medical Center under IRB, protocol no 0043-22-EMC. From these specimens, we excluded patients with synchronous tumors, patients with insufficient quantities of tissue for further archival preservation, and patients who received therapies prior to surgery. Tissue cores were obtained from the original FFPE tissue blocks and from tissue stocks kept in the Pathology Department of Emek Medical Center. All tissue blocks were constructed by using a TMA grand master system (3DHistech Ltd., Budapest, Hungary). Each case underwent precise evaluation by a pathologist to identify the tumor from which the core could be extracted. Up to 60 2 mm cores were placed in each TMA block for a total of six TMA blocks. The first 4-μm section from each block was used for hematoxylin and eosin staining. All patient data was unavailable at the time of experimental analysis to ensure non-biased analysis, and as such was blinded to the experimenter.

### Immunofluorescent staining of human tumor microarray

Human TMAs containing samples from patients were deparaffinized and fixed with 10% neutral buffered formalin. Antigen retrieval was performed using citrate buffer (pH 6.0). Slides were then blocked with 10% BSA + 0.05% Tween20 and the antibodies were diluted in 2% BSA in 0.05% PBST and used in a multiplexed manner with OPAL reagents (Akoya Biosciences). All primary antibodies (described in Supplementary Table S4) were incubated overnight at 4 °C (1:400) according to the following staining sequences: Set 1: KLC1 → KIF5B → CK → DAPI; Set 2: EIF4G2 → CK → DAPI. Each antibody was validated and optimized separately and then the multiplex staining was optimized. Briefly, following primary antibody incubation, slides were washed with 0.05% PBST, incubated with secondary antibodies conjugated to HRP (1:400), washed again, and incubated with OPAL reagents. Slides were then washed, and antigen retrieval was performed. Then, slides were washed with PBS and stained with the next primary antibody or with DAPI at the end of the cycle. Finally, slides were mounted using Immu-mount (#9990402, Thermo Scientific). Images were taken with a Phenocycler scanner (Akoya®) and analyzed using QuPath software. Cell segmentation was done using Cellpose. For each patient, average cell intensities for the indicated markers was calculated, and correlation between the different markers was analyzed.

### Survival analysis

Patients were stratified based on different parameters (tumor grade, stage and type, and high/low staining intensities of EIF4G2, KIF5B and KLC1, compared to median expression levels for each). Kaplan–Meier (KM) analysis of OS and RFS with log rank P value was performed on patients stratified by these parameters. Paired comparisons were calculated using the Survdiff function in R with FDR correction.

### Cell lines and cell culture

Authenticated HEC-1A (HTB-112) and RL95-2 (CRL-1671) cells obtained from the ATCC (in 2020) were used at early passage number and routinely checked for mycoplasma contamination. Cells were cultured in DMEM/F-12 (Gibco 21331-020) medium with 10% FBS (Gibco 12657-029), 1% penicillin streptomycin (Biological Industries 03031-1B) and 1% L-Glutamine (Biological Industries 03-020-1B). Stable KD of EIF4G2 was generated by infecting HEC-1A or RL95-2 cells with lentiviruses harboring pLKO.1-puro plasmid expressing shRNA targeting GFP (Control) or shRNA targeting EIF4G2 (Sigma TRCN0000147914), followed by selection using puromycin, as previously described [14]. Transient EIF4G2 KD was generated by transfecting HEC-1A cells with control siRNA (Dharmacon D-001810-10) or siRNA targeting EIF4G2 (Dharmacon L-011263-00-0020) using JetPEI transfection reagent (Polyplus 101000020) according to manufacturer’s protocol.

For co-culturing experiments, control HEC-1A or RL95-2 cell were stained with CFSE (BioLegend, 750001883, 1:20000) for 5 min on ice, followed by extensive washes with PBS. 0.5 × 10^6^ stained control cells were mixed and seeded with equal amounts of EIF4G2KD cells. 24 h following seeding, cells were subjected to 8 (RL95-2 cells) or 16 Gy (HEC-1A cells) X-Ray irradiation using XRAD 320 (Precision X-Ray) or mock-irradiated for control, and 72 h later subjected to flow cytometry analysis.

### Cell viability assay

Cells were plated in sealed white 96-well plates (5000 cells/well). Luminescence was measured 24 h after plating (T0) and after 4 d using the CellTiter-Glo Luminescent Cell Viability Assay protocol (Promega; cat# G7573). When indicated, cells were treated 24 h after plating with 2.5 nM Taxol (Paclitaxel; Sigma-Aldrich; cat# 7402) or subjected to 8 or 16 Gy X-Ray irradiation. Control cells were subjected to incubation with DMSO or mock irradiation, respectively. CellTiter-Glo luminesce values of treated cells were normalized to that of control cells 4 d after treatment to obtain the relative viability.

### Flow cytometry analysis and sorting

For cell cycle analysis, cells were stained in culture with Hoechst 33342 (Sigma-Aldrich, H3570, final conc. 1 µg/mL) for 1 h at 37 °C. Cells were then trypsinized, collected and incubated with PI (Sigma-Aldrich, P4864), to assess cell viability. For cell surface marker expression analysis, 0.5–2 × 10^6^ cells were trypsinyzed, collected and washed. Cells were then stained with anti CD133-APC conjugated antibody ((Thermo Fisher Scientific Cat# 17-1338-42, RRID:AB_1603199), 1:100) or CD44-Alexa Fluor 700 ((Thermo Fisher Scientific Cat# 56-0441-82, RRID:AB_494011), 1:400) for 30 min at 4 °C. Cells were counterstained with PI to assess viability. Data acquisition was performed on CytoFLEX flow cytometer and software (Beckman Coulter Life Sciences) and data analysis was performed using FlowJo software version 10.8.1.

Control and EIF4G2KD HEC-1A cells were separated using magnetic beads for CD133 (CD133 Microbead Kit, Miltenyi Biotec, 130-097-049), or CD44 (CD44 Microbeads Kit, Miltenyi Biotec, 130-095-194) for RL95-2 cells, on LS-columns (Miltenyi Biotec, 130-042-401) according to the manufacturer’s recommendations. For RNA-seq and mass spectrometry analysis, separated control and EIF4G2KD HEC-1A cells were stained with PI and CD133-APC conjugated antibody, sorted by BD LSRII flow cytometer using Diva software (BD Biosciences), and collected and pelleted for further use.

### MARS-seq library preparation and data analysis

15 000 CD133+ were FACS sorted into 40 μL of lysis binding buffer and mRNA was isolated using Dynabeads mRNA DIRECT Purification Kit (Thermo-Fisher scientific; cat# 61012) according to manufacturer’s protocol. MARSseq Libraries were generated by the MARS-seq protocol [47]. The analysis of the MARS-seq data with the UTAP (User-friendly Transcriptome Analysis) Pipeline, alignment to hg38 with STAR v2.4.2a, normalization and differential expression analysis by DESeq2 were all performed as previously described [48]. Raw *P* values were adjusted for multiple testing using the procedure of Benjamini and Hochberg. Genes with baseMean expression >10, log2FC > 0.58 (fold change >1.5), and p.adj <0.05 were considered as genes with differential expression.

### Mass spectrometry

Sorted cell pellets were lysed in 50 mM Tris-HCl pH 7.4, 5% SDS, and sonicated (Bioruptor Pico, Diagenode, USA). Protein concentration was measured using the BCA assay (Thermo Scientific, USA). From each sample, 20 μg of total protein was subjected to in-solution tryptic digestion using the suspension trapping (S-trap) method as previously described [49]. Liquid chromatography and Mass Spectrometry was performed as described previously [24], using split-less nano-Ultra Performance Liquid Chromatography, reversed-phase Symmetry C18 trapping column (Waters) and a T3 HSS nano-column (Waters) for desalting and separation, coupled to a quadrupole orbitrap mass spectrometer (Q Exactive HFX, Thermo Scientific). Data acquisition, processing and analysis was performed as described [20]. Differential protein abundance was tested using the glht R function by ANOVA followed by Tukey analysis on the intensity values using a logarithmic scale. Changes in protein abundance of at least 1.5 between conditions, with *p*.value < 0.05 were considered significant.

### Western blot analysis

Cells were lysed in RIPA lysis buffer and subjected to western blotting as previously described [24], using antibodies listed in Supplementary Material- antibody list. Western blot results were imaged using ImageJ software. Quantification of protein steady levels was performed and normalized to the appropriate loading control and log transformed.

### Polysomal profiling

Control and EIF4G2KD HEC-1A CD133+ and CD133-fractionated populations were subjected to polysomal fractionation as described [14].

### Statistical analysis

All additional statistical analyses not described above were performed using Prism 9.3 software (GraphPad Software), as specified in figure legends. F-tests were performed to confirm equal variance among samples. Non-significant comparisons were mostly not marked in the graphs.

### Supplementary information

Supplementary Material

Supplementary Table S1

Supplementary Table S2

Supplementary Table S3

## Data Availability

The mass spectrometry datasets generated during and/or analyzed during the current study are available in the MassIve repository of the ProteomeXchange consortium (https://massive.ucsd.edu), with the dataset identifier MSV000092778. The RNA-seq datasets generated during and/or analyzed during the current study are available in the Gene Expression Omnibus (GEO) at GSE242717. All other data generated or analyzed during this study are included in this published article and its supplementary information files.
